# Quality of analgesia in physician-operated telemedical prehospital emergency care is comparable to physician-based prehospital care - a retrospective longitudinal study

**DOI:** 10.1038/s41598-017-01437-5

**Published:** 2017-05-08

**Authors:** Niklas Lenssen, Andreas Krockauer, Stefan K. Beckers, Rolf Rossaint, Frederik Hirsch, Jörg C. Brokmann, Sebastian Bergrath

**Affiliations:** 1 Department of Anaesthesiology, University Hospital RWTH Aachen, Pauwelsstr. 30, 52074 Aachen, Germany; 2 Emergency Medical Service, Fire Department, City of Aachen, Stolberger Str. 155, 52068 Aachen, Germany; 3 Emergency Department, University Hospital RWTH Aachen, Pauwelsstr. 30, 52074 Aachen, Germany

## Abstract

Acute pain is a common reason for summoning emergency medical services (EMS). Yet in several countries the law restricts opioid-based analgesia administration to physicians. Telemedical support of paramedics is a novel approach to enable timely treatment under the guidance of a physician. In this retrospective observational study, conducted in the EMS of Aachen, Germany, the analgesic quality and occurrence of adverse events were compared between telemedically-supported paramedics (July-December, 2014) and a historical control group (conventional on-scene EMS physicians; January-March, 2014). Inclusion criteria: pain (initial numerical rating scale (NRS) ≥5) and/or performed analgesia. Telemedically-assisted analgesia was performed in 149 patients; conventional analgesia in 199 control cases. Teleconsultation vs. control: Initial NRS scores were 8.0 ± 1.5 and 8.1 ± 1.7. Complete NRS documentation was carried out in 140/149 vs. 130/199 cases, p < 0.0001. NRS scores were reduced by 4.94 ± 2.01 and 4.84 ± 2.28 (p = 0.5379), leading to mean NRS scores at emergency room arrival of 3.1 ± 1.7 vs. 3.3 ± 1.9 (p = 0.5229). No severe adverse events occurred in either group. Clinically relevant pain reduction was achieved in both groups. Thus, the concept of remote physician-based telemedically-delegated analgesia by paramedics is effective compared to analgesia by on-scene EMS physicians and safe.

## Introduction

Acute pain is a frequent and relevant symptom in prehospital emergency medical care. While 20–31% of all patients being transported by emergency medical services (EMS) indicate experiencing moderate to severe pain, 35–70% of trauma patients experience pain in prehospital settings^1–3^. Early and sufficient prehospital analgesia can prevent negative pain-related physiological and psychological effects, helps to facilitate transportation as well as therapeutic manoeuvres, and is mandatory for ethical reasons^4–7^. Different guidelines (e.g., major trauma and acute coronary syndrome) recommend sufficient prehospital pain reduction^1, 8–10^. Pain is commonly measured with the numerical rating scale (NRS, range 0–10)^11^. A reduction in pain (ΔNRS) of ≥2 or a NRS score ≤5 at the end of the mission is clinically defined as adequate reduction of pain severity, based on a published and internationally used (minimum) standard^12–15^. Nevertheless, up to 50–90% of all patients receive insufficient pain therapy in several western countries, such as the Netherlands, Switzerland and the USA^3, 4, 16^.

While many countries run EMS solely with paramedics, legal conditions in several countries restrict opioid-based analgesia administration to physicians. This is one reason, why some countries (e.g., Germany) run two-tiered emergency response systems involving both ambulance units staffed by paramedics and units staffed with prehospital EMS physician. Based on local protocols regional emergency dispatch centres deploy both kinds of emergency units. According to the recommendations of the German Medical Association, an EMS physician should be dispatched in all potentially life-threatening situations and severe pain conditions^17^.

Due to both dispatch and staffing reasons, paramedics are often the first to arrive on-scene, and a substantial amount of time may pass before an EMS physician unit can arrive. Therefore, certain treatments like intravenous analgesia are often delayed.

In recent years, telemedicine has emerged as a complementary system in EMS, which may provide remote medical expertise and sustain or even improve the quality of medical treatment provided on-scene. Previous research has demonstrated positive impacts of telemedical systems on treatment processes and even patient outcomes^12, 18–23^.

A comprehensive simulation study revealed that telemedically-supported paramedics were able to perform advanced treatments in simulated emergencies with a quality of care comparable to that provided by on-scene EMS physicians^24^. A previous pilot study from the research project “TemRas” (telemedical rescue assistance system, 2012–2013) compared the safety and quality of analgesic treatment between telemedically-supported paramedics and on-scene EMS physicians in different EMS districts. These results showed that both systems of care (i.e., analgesia by on-scene EMS physicians and by telemedically-supported paramedics) are safe and provide analgesia above the required minimum standard in a multicentre research project setting, but analgesia by on-scene physicians led to higher pain score reduction^12^.

Moreover, a comprehensive telemedicine system provides legal certainty for paramedics in previously trained treatment cases (e.g. opioid administration) according to German law, even without the physical presence of a pre-hospital emergency physician^25, 26^.

Based on these findings, the concept of telemedical support for paramedics was implemented into EMS routine care beginning April 2014 in the EMS district of Aachen, Germany. The German health insurance companies fund this system in Aachen as a pilot region. Therefore, the analgesic treatment piloted in the research project, and which was defined in a standard operating procedure (SOP), was extended and implemented in routine EMS.

To evaluate the quality of analgesia and the incidence of severe adverse events in telemedically-supported emergency care treatment by paramedics, data were compared to a historical conventional treatment group, which consisted of analgesia performed by on-scene EMS physicians.

## Methods

### Study setting

Teleconsultation protocols and paper-based EMS physician protocols were screened for patients reporting pain levels with NRS scores ≥5 and/or documented analgesic treatment. The investigated time period was chosen due to the implementation of a physician-based telemedical service into routine clinical practice in the Aachen EMS system on April 1^st^, 2014. By default, analgesia was performed by prehospital emergency physicians until March 31^st^, 2014 due to legal regulations. The teleconsultation system offers year-round support for paramedics, who are at an emergency site and en route to a hospital during any type of emergency. Initially, this new service operated daily from 07:30 a.m. to 08:15 p.m. From July 1^st^, 2014 on it was extended to a round-the-clock coverage system.

To avoid possible interference between these complementary systems, data from a historical group were retrieved to serve as the control (i.e., January 1^st^, 2014 - March 31^st^, 2014). During this time span, no telemedical support was available.

The study uses the STROBE guidelines for case-control studies^27^.

### Ethics statement and data privacy

Each patient gave verbal consent for teleconsultation prior to the start of data transmission. The study was approved by the institutional ethics committee (University Hospital RWTH Aachen, Germany, registration number EK 109/15) and data analysis was performed after pseudonymisation for both groups. Therefore informed consent for data analysis was waived by the ethics committee.

### Trial registration

This trial was registered as a retrospective study at www.clinicaltrials.gov (NCT02928705).

### Telemedicine system

Telemedical support was performed upon request of on-scene paramedics, based on a predefined standard operating procedure (SOP) for different emergency situations (i.e., analgesic treatment in trauma and non-trauma situations).

Using a mobile and an in-car data transmission unit (peeqBox, P3 telehealthcare GmbH), encrypted data and audio transmission was established to provide the following functionalities: two-way audio connection, real-time vital data transmission (numerical values and curves), still picture transmission, 12-lead-electrocardiogram (ECG) transmission, and real-time video streaming from the inside the ambulance. A detailed description of the technical system is published elsewhere^28^. On the telemedical workstation, real-time vital data and ECGs were displayed using commercial software (corpuls.web, GS Elektromedizinische Geräte, Kaufering, Germany). All other data were displayed on telemedical software that enabled context-sensitive algorithms and checklists (Telemedical Documentation, P3 telehealthcare, Aachen, Germany).

**Figure 1:**
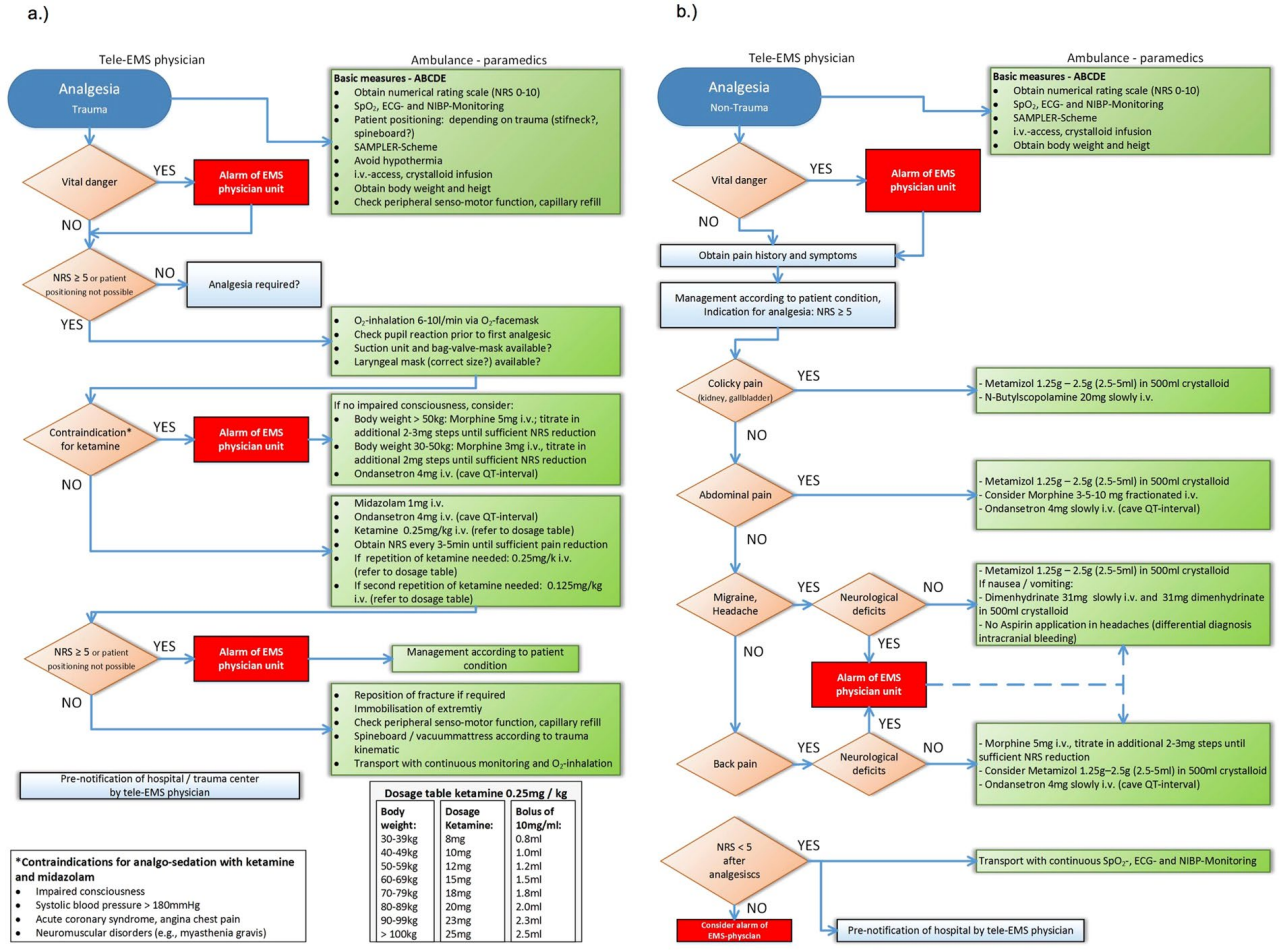
Standard operating procedure (SOP) for analgesia in teleconsultation: (**a**) trauma, (**b**) non-trauma.

When analgesia was required, the SOP that had been taught to EMS personnel could be displayed on the workstation in the teleconsultation centre to support the tele-EMS physician in therapy recommendations (Fig. 1). Standardised teleconsultation protocols were generated by the tele-EMS physician for each patient. A hard copy was available for the tele-EMS physician, the paramedics (printer inside the ambulance), and for the handoff at the admitting hospital.

### Selection of participants

Teleconsultation and EMS physician protocols were screened for patients with initial NRS scores ≥5 and/or analgesic treatment.

Patient data were excluded for any of the following reasons:

1. Missing consent for telemedical consultation (teleconsultation group).
2. Analgesia in non-ST-segment elevation acute coronary syndrome (Non-STEMI-ACS) and ST-elevation myocardial infarction (STEMI).
3. Initially unconscious patient.
4. Inter-hospital transfer mission.
5. Cases involving both on-scene and telemedical EMS physicians, as their respective influence on prehospital analgesia could not be evaluated.

The following restrictions applied to the teleconsultation group independently from the study setting due to German regulations: a physically present EMS physician on-scene is obligatorily required in all life-threatening emergency cases (e.g. severe trauma, clouding of consciousness, haemodynamic instability). In such cases, the teleconsultation system could be used as bridging support until arrival of an EMS physician on-scene and/or as support for an on-scene EMS physician. Such data are not be presented in this study due to exclusion criteria 5.

**Figure 2:**
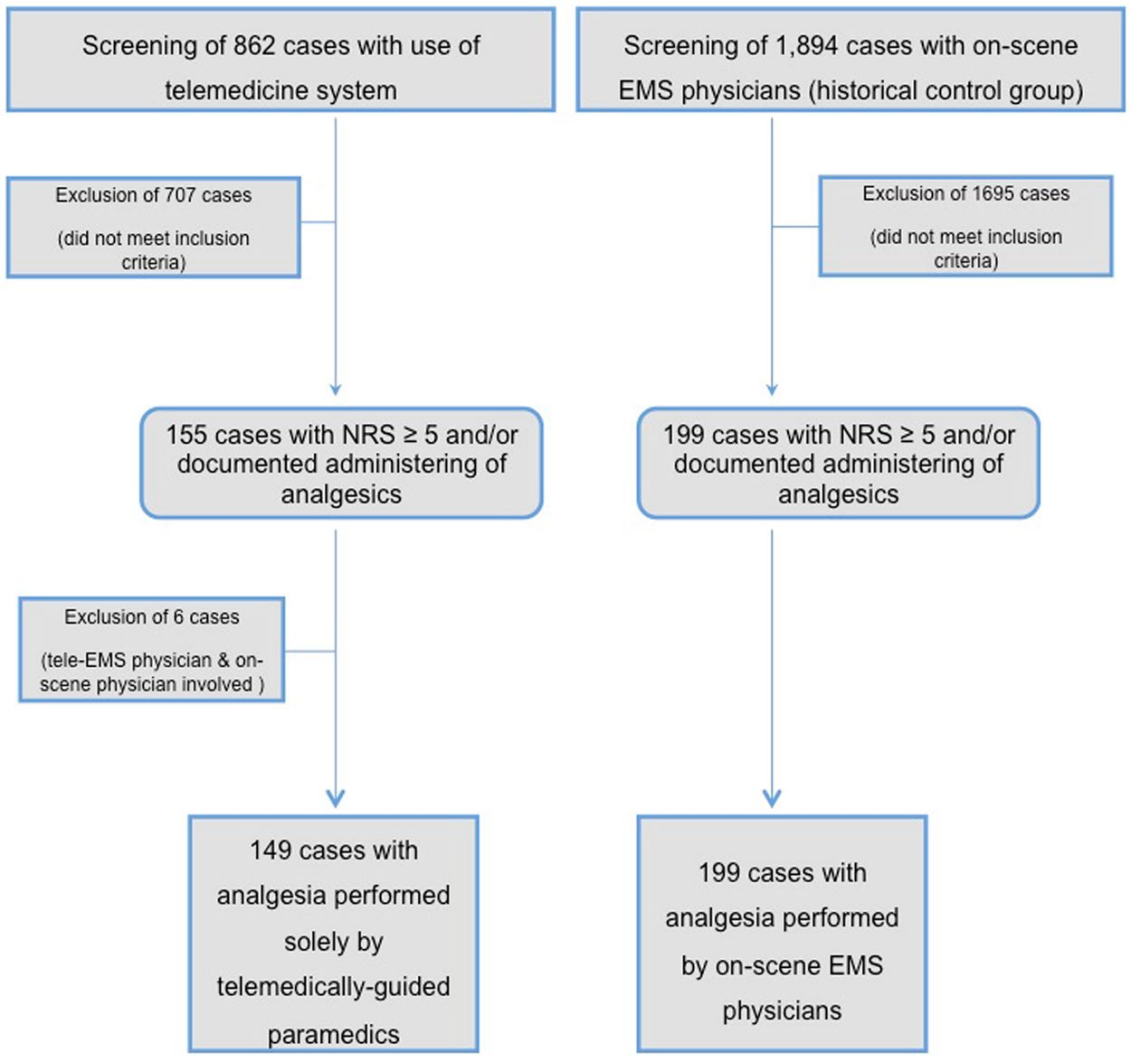
Study flow chart.

The study flow chart is presented in Fig. 2.

### Outcomes and data sources

Analgesic quality was the primary outcome measure and was operationalized using the NRS, which was applied by default during initial contact with the patient and at the end of the mission. Pain score reduction (ΔNRS) was calculated to compare both groups.

Secondary outcome measures were the rate of intervention-related severe adverse events, the occurrence of nausea and vomiting, the administered medications, as well as their dosages and influence on vital signs. The analysis of severe adverse events focussed on respiratory insufficiency (e.g., immediate treatment-requiring decline in oxygen-saturation, apnoea), circulatory insufficiency (immediate treatment-requiring hypotension/hypertension, circulatory arrest), severe allergic reactions (immediate administration of medication (e.g., antihistamines, adrenaline) required), other life-threatening situations, death.

Data on patient demographics, prehospital diagnoses, severity of illness/injury using the National Advisory Committee on Aeronautics severity (NACA) score, prehospital treatment, and medication administration were retrieved from the EMS and teleconsultation protocols. Time intervals were extracted from the protocols and from EMS dispatch centre data.

### Statistical analysis

Ordinally scaled parameters (e.g., NRS, NACA) were compared using the Mann-Whitney U test. Continuous variables (e.g., systolic blood pressure, age) were first tested with the Kolmogorov-Smirnov test for standard distribution. In case of normally distributed data, an unpaired t-test without Welch correction was carried out. Whenever data were not distributed normally, the Mann-Whitney U test was conducted. Contingency tables were analysed using the Chi-squared test. All statistical analyses were performed using GraphPad Prism 7.0 (GraphPad Software, La Jolla, CA, USA). Due to the exploratory nature of the study, p-values < 0.05 were considered to be significant.

## Results

### Study participants

During the six-month study phase, teleconsultation was performed in 862 emergency missions. Of these, 155 patients (18%) met the inclusion criteria. Cases of teleconsultation with complementary analgesic treatment by an on-scene physician were excluded from the analysis (n = 6). Thus, there were 149 cases where the paramedics administered analgesia with support from the tele-EMS physician (Fig. 2).

In the historical control group, prehospital EMS physicians treated 1,894 patients on-scene between January 1^st^, 2014 and March 31^st^, 2014. Of these, 166 patients had an initial NRS score ≥5. In 33 cases no initial NRS score was documented, but analgesia treatment was performed. Therefore, 199 (11%) patient data sets were eligible for the control group in the study (Fig. 2).

Patient characteristics and demographics for the study and control groups are summarized in Table 1.

**Table 1:** Patient characteristics and demographics.

	Telemedicine group	Historical control group	p-value
Number of patients	149	199	
Female (%)	57.1	53.3	0.5151
Age (years)	57.1 ± 22.8	55.5 ± 25.2	0.5571
	n = 146	n = 198	
Trauma (%)	43.6	48.2	0.3927
NACA-Score	3.1 ± 0.4	3.2 ± 0.5	0.0129
	n = 149	n = 176	
Initial value on numerical rating scale (0–10 points)	8.0 ± 1.5	8.1 ± 1.7	0.3843
	n = 149	n = 166	

(SD = standard deviation; CI = Confidence Interval). Parameters are displayed as numbers, percentages and means ± standard deviation.

### Analgesic quality

The ΔNRS was calculable in 140/149 cases in the telemedicine group (94%) vs. 130/199 in the control group (65%), p < 0.0001; in the remaining cases, ΔNRS could not be calculated due to incomplete NRS documentation. No significant difference in numerical reduction in NRS score between both groups was found: 4.94 ± 2.01 (telemedicine) versus 4.84 ± 2.28 (control), p = 0.5379. The average initial NRS scores were reduced to 3.1 ± 1.7 (telemedicine) and 3.3 ± 1.9 (control) at the time of handoff at the emergency department, p = 0.5229.

### Analgesics

Morphine was the most frequently used opioid analgesic in the telemedicine group (80/149), whereas piritramide was the most common opioid analgesic in the control group (67/199). All of the applied analgesics, their frequency of application, and the dosages used are summarized in Table 2. While the usage rates of the respective analgesics differed statistically significant between the groups, the respectively used dosages did not, with the exception of ketamine (Table 2).

**Table 2:** Type, frequency, and dosages of administered medications.

Medication used dosage (mg)	Telemedicine group n = 149	Control group n = 199	p-value
Morphine	n = 80 (53.7%)	n = 18 (9.1%)	<0.0001
	5.7 ± 2.0 mg	5.5 ± 4.2 mg	0.8459
Piritramide	n = 4 (2.7%)	n = 67 (33.7%)	<0.0001
	5.8 ± 2.3 mg	7.7 ± 4.9 mg	0.2047
Fentanyl	N.A.	n = 57 (28.6%)	N/A
		0.2 ± 0.1 mg	
Metamizol	n = 83 (55.7%)	n = 60 (30.2%)	<0.0001
	2395 ± 354 mg	2200 ± 800 mg	0.0812
Ketamine	n = 33 (22.2%)	n = 25 (12.6%)	0.0176
	32.88 ± 17.63 mg	52.8 ± 31.53 mg	0.0074

(Dosage in mg ± SD; SD = standard deviation).

### Adverse events

Severe adverse events occurred in neither group (p = N/A). In the telemedical group, one case of moderate hypotension (systolic blood pressure = 86 mmHg) occurred after administering midazolam and ketamine, but was immediately stabilized after delegated intravenous application of cafedrinhydrochloride/theodrenalinhydrochloride. Two cases of local rash after metamizol administration were reported (telemedicine group). In one case, medication administration was immediately stopped and only crystalloid fluid was infused. In the second case, an immediate and complete relief of the rash was described. For both cases, no systemic reaction was observed such that no other intervention was required.

In the historical control group, one case of vertigo was observed after administering fentanyl. No intervention was needed. Nausea was detected in 27 patients (18.1%, telemedical group) and 20 patients (10.1%, control group) prior to analgesic treatment. After analgesic treatment, one additional case (0.7%) of nausea was reported in the telemedical group versus three cases (1.5%) in the control group. Vomiting was observed once (0.7%) in the telemedical group and twice (1.0%) in the control group. Antiemetic therapy was administered in 93 patients (62.4%) in the telemedical group and in 96 patients (48.2%) in the control group.

A comparison of vital signs before analgesic treatment and at the time of patient handoff is presented in Table 3, as are their rates of documentation.

**Table 3:** Analgesia-related safety parameters before and after analgesic treatment at the time of handoff at the emergency department.

	Telemedicine group (n = 149)	Historical control group (n = 199)	p-value
Initial heart rate (beats/min)	83.7 ± 17.4 (83)	90.7 ± 21.1 (89)	0.0017
	n = 132 (88.6%)	n = 196 (98.5%)	
Heart rate (beats/min) at handover	82.8 ± 15.8 (80.5)	87.1 ± 16.3 (88.5)	0.0181
	n = 146 (98.0%)	n = 170 (85.4%)	
Initial non-invasive systolic blood pressure (mmHg)	151.3 ± 27.7 (149)	142.8 ± 29.6 (140)	0.0077
	n = 145 (97.3%)	n = 189 (95.0%)	
Non-invasive systolic blood pressure (mmHg) at handover	143.8 ± 23.5 (141)	134.9 ± 23.1(130)	0.0011
	n = 148 (99.3%)	n = 156 (78.4%)	
Initial non-invasive diastolic blood pressure (mmHg)	88.2 ± 16.6 (88)	87.1 ± 19.2 (81)	0.6902
	n = 132 (88.6%)	n = 116 (58.3%)	
Non-invasive diastolic blood pressure (mmHg) at handover	85.1 ± 15.8 (84)	80.1 ± 13.6 (80)	0.0317
	n = 142 (95.3%)	n = 94 (47.2%)	
Initial oxygen saturation (%)	97.5 ± 2.5 (98)	97.1 ± 3.4 (98)	0.2963
	n = 142 (95.3%)	n = 196 (98.5%)	
Oxygen saturation (%) at handover	97.9 ± 2.0 (98)	97.1 ± 7.1 (98)	0.2916
	n = 148 (99.3%)	n = 169 (84.9%)	
Initial value on Glasgow Coma Scale	15.0 ± 0.2 (15)	14.9 ± 0.5 (15)	0.0305
	n = 138 (92.6%)	n = 186 (93.5%)	
Value on Glasgow Coma Scale at handover	15.0 ± 0.0 (15)	14.7 ± 1.2 (15)	0.0123
	n = 136 (91.3%)	n = 181 (91.0%)	
Initial respiratory rate	14.8 ± 2.1 (14)	16.7 ± 3.6 (16)	0.0007
	n = 48 (32.2%)	n = 137 (68.8%)	
Respiratory rate at handover	14.5 ± 2.6 (14)	15.1 ± 2.5 (14)	0.1233
	n = 68 (45.6%)	n = 116 (58.3%)	

Telemedical group versus control group. Parameters displayed as means ± standard deviation and medians in brackets.

Of the six cases that involved both a tele-EMS physician and an on-scene physician (excluded from the study), the reasons for requiring both units included:

- The patient’s wife worked as an EMS physician: after consulting with the tele-EMS physician and during constant supervision by the tele-EMS physician, the patient’s wife administered analgesics (n = 1).
- An on-scene physician was requested when the paramedics experienced difficulties inserting an intravenous line (n = 1).
- An on-scene physician was initially dispatched as the emergency dispatch centre expected a life-threatening case. However, a life-threatening situation was not detected on-site, thus the EMS physician started analgesic treatment and handed over the case to the tele-EMS physician (n = 2).
- An on-scene physician was dispatched additionally as inadequate pain reduction was achieved, despite telemedically-guided administering of analgesics by paramedics (n = 2).

### Duration of treatment

The mean duration of teleconsultation, defined as the time period from the beginning of teleconsultation to the completion of the teleconsultation protocol was 36.5 ± 16.0 min. In comparison, cases with on-scene EMS physician care lasted 41.3 ± 17.9 min, p = 0.01.

## Discussion

Telemedically-delegated analgesia led to adequate pain reduction^15^, that was comparable to reductions obtained by prehospital EMS physicians. Overall, teleconsultation was found to be safe, and only minor complications due to analgesia were observed in both groups. Moreover teleconsultation enables physicians to treat patients in non-life-threatening cases is a shorter time and in a wider radius.

To the best of our knowledge, this is the first study investigating the quality of analgesia in trauma and non-trauma patients and the incidence of adverse events in a physician-based telemedical service in emergency routine care.

Limited human and financial resources constrain the ability to provide the best possible treatment for each patient. Therefore, a strategic deployment of rare resources (e.g., qualified prehospital EMS physicians) is needed and alternative concepts of emergency care are required in the future. However, in several countries (e.g., Germany) legal conditions restrict opioid-based analgesia administration to physicians. New concepts (i.e., teleconsultation) based on research findings need to be evaluated in daily routine care in order to ensure timely and adequate treatment in urban, and particularly in rural areas.

In this study, the analgesic quality of telemedically-guided therapy was comparable to on-scene physician care. In addition, both groups achieved adequate pain reduction^15^. Demographics, NACA severity score, initial NRS, and pain reduction are clinically comparable in both groups. A statistically significant difference in the solely descriptive and not validated NACA severity scores (3.1 ± 0.4 (teleconsutation group), 3.2 ± 0.5 (historical control group), p = 0.0129) is assumed to be not significant for medical treatment. Thus, these findings support the thesis that analgesia administration in non-acute life-threatening situations can be carried out solely by telemedically-supported paramedics without requiring an on-site physician. The quality of care was not affected negatively by the use of telemedicine. Although a definitive safety assessment in telemedically-delegated analgesia is not possible due to the reported sample size, recently-published results from the precursor research project as well as scientific data for prehospital analgesia in general support the thesis that telemedically-based prehospital analgesia is a safe procedure^12, 13^.

In contrast to the study setting, some countries run EMS solely with paramedics. Furthermore, the legal restrictions of administering opioids may vary in different regions or countries. Hence, it is debatable whether paramedics could perform (opioid-based) analgesia with the same quality as EMS physicians or telemedically-supported paramedics without supervision by a physician. There is evidence that application of analgesics by specially trained paramedics is possible. Middleton *et al*. described methoxyflurane, fentanyl and morphine as effective agents in prehospital analgesia by paramedics^29^. Although methoxyflurane was described as the least potent analgesic in that study, Bendall *et al*. found it to be the most commonly administered agent in combination with other non-opioid agents^30^. A study by Walsh *et al*. revealed that paramedics had several reasons not to administer opioids or to administer too low dosages. There was a reluctance to administer opioids to patients without significant objective signs for pain, a preoccupation with potential malingering, an ambivalence about the degree of pain control to target or to expect, a fear of masking diagnostic symptoms, and an aversion to aggressive dosing of opioids^31^. To achieve higher rates of adequate pain reduction, it seems to be sensible to offer telemedical support for paramedics, even if they are legally authorized to administer opioids.

A study by Reimann *et al*. points out that the increasing number of EMS cases over recent years causes a relevant capacity utilisation of on-scene EMS physicians, so that an immediate availability of an EMS physician could not be ensured in all cases^32^. Schmiedel *et al*. indicate that the time interval from alerting EMS physicians until arrival on-scene has significantly increased over the last years, so that some regions (e.g., Baden-Württemberg, Germany) have even considered extending the current legal time frame in which EMS-based medical aid must be provided^33^.

This is a setting in which the teleconsultation system could be very beneficial. In our current study, the mean mission duration differed statistically significant between the groups. Telemedically-delegated analgesia does not slow down the treatment process. This is also noteworthy as in teleconsultation cases only half of the usual staff is on-site (two paramedics versus one on-site EMS physician, and three paramedics). Another advantage of teleconsultation is that it eliminates the need for driving time to and from the place of emergency for EMS physicians. Thus, teleconsultation can support more patients in a shorter time and in a wider radius.

In the current study, a SOP was only implemented for the telemedicine group. The higher heterogenity in administered medications in the control group can be explained by the lack of a SOP. While morphine was the only opioid in the teleconsultation SOP, prehospital EMS physicians had morphine, piritramide, and the more potent fentanyl available. Considering the fact that demographics, NACA severity score, initial NRS, and pain reduction are clinically comparable in both groups, the medication regimens could be explored in future studies. Available medication (e.g., single versus multiple opioid disposability) and medication combinations (e.g., morphine and ketamine) need further evaluation in terms of analgesic effectiveness, patient comfort, and safety in the prehospital telemedical emergency care setting. Moreover, alternative routes of medication administration (e.g. intranasal application) must be analysed in the future^34, 35^. The current study gives initial indications that analgesic treatment based on simple algorithms and with a small list of medications might achieve similar analgesic quality compared to the use of multiple analgesics. Future studies should investigate this aspect further, including the question whether simplicity could help improve patient safety and reduce contingency costs.

Brokmann *et al*. reported data from the precursor research project^12^. In that study, the extent of pain reduction in the telemedicine setting was sufficient but not as high as with analgesia treatment by on-site EMS physicians. In contrast, in the current study we observed comparable pain reduction in both groups with implementation of the procedure into routine care. There are several possible reasons for this difference. In terms of the research project, the telemedicine system represented a completely new and therefore challenging role for both the paramedics and the tele-EMS physicians. A new system in which responsibility for patients occurs remotely is a probable reason for the lower medication dosages observed and consequently the lower pain reduction obtained in the Brokmann *et al*. study. Additionally, the SOP for telemedical analgesia used during the research project was more restrictive, with fewer medications and lower dosages. Friesgaard *et al*. recently described similar effects when launching intravenous opioid administration by non-physician ambulance personnel in general^13^. Moreover, our inclusion criteria differed slightly. Pain management for acute coronary syndrome was excluded from this investigation, as simultaneous treatment with other medications such as acetylsalicylic acid and nitroglycerin may have impeded comparability with other cases of pain management. Therefore, it might be reasonable to compare the efficacy and safety of different analgesia approaches for specific indications in general in the future, and in particular for settings in which medication administration is delegated (e.g., telemedicine).

The incidence of nausea and vomiting after analgesic treatment was low and occurred to a clinically similar extent in both groups. Vital signs were not affected negatively in either group. Although statistically significant differences were found between both groups for some vital signs, these were deemed not clinically relevant in these emergencies.

For patient safety reasons and medicolegal reasons, high quality documentation is required. The telemedical setting led to a significantly higher integrity of documentation for the primary outcome parameter (documentation of NRS at two defined points in time) compared to the control group (94% versus 65%, p < 0.0001). As seen in Table 3, many other quality and safety parameters relevant to patient monitoring were frequently documented in both groups. In the teleconsultation group, especially at the time of handoff to hospital staff, documentation rates were higher. Nevertheless, the telemedicine group in particular showed low rates of respiratory rate documentation. However, although there is the potential to improve the quality of documentation in both study groups, the quality of documentation found in both study groups was higher than that found by Bloemhoff *et al*. in the Dutch EMS^36^. An inclusion of solely well-documented cases in this study would have led to an unintended selection bias. To prevent this bias, we have analysed all cases with analgesic treatment and not only those with an explicitly documented NRS score ≥5.

Assessing the technical performance of the telemedicine system was not the objective of this study, but there were no reports of relevant interference (e.g., disconnection of audio or vital data) that could have troubled telemedical care. In general, the described system is a very reliable and stable system, as reported by Felzen *et al*.^28^.

## Limitations

Time-dependent confounders might have reduced the comparability of these data. Seasonal differences in number and type of trauma as well as illnesses are known. But also changes in therapy guidelines, in medical equipment, and in medical staff might affect study results. In order to minimize the effect of potential confounders, we analysed two different time periods in one year: data for the conventional care group were extracted from the first quarter of 2014, whereas data for the teleconsultation group were taken from the time of implementing a round-the-clock coverage teleconsultation system (July 1^st^, 2014) until December 31^st^, 2014.

We anticipated we would observe a systematic impact on the treatment behaviour of on-scene emergency physicians after implementing the highly standardized teleconsultation system. In order to avoid this influence of the teleconsultation system on on-scene emergency physicians, we deliberately chose to use a historical control group. Furthermore, fewer EMS physician missions (e.g., for analgesia) were required after implementation of the telemedicine system. Therefore, different patient collectives for on-scene EMS physicians are probable in the historical control group and in the conventional treatment group during the phase where teleconsultation was available. In this pilot study, no formal sample size calculation and power analysis was possible. All results must be interpreted against this backdrop and retrospective design, which has general limitations.

For EMS settings, scientific data measuring quality in pain control are rare. The applied minimum standard^15^ was also used within the scope of the telemedical research project TemRas^12^ and in similar extent in recent international studies evaluating the efficacy and safety of intravenous opioid administration by ambulance personnel and in analgesic treatment of paediatric patients^13, 14^.

Blinding during analysis was not possible due to different layouts of the original documentation protocols. Therefore, detection bias of the secondary endpoint adverse events has to be taken into consideration. Furthermore, we cannot rule out the possibility that patients might have developed adverse events after arriving in the hospital due to prehospital treatment. However, these possible limitations apply equally to both groups.

In two cases an on-scene physician had to be dispatched secondarily, as insufficient pain reduction was achieved by telemedically-guided administration of analgesics. These two patients were excluded from the study, as the influence of the respective treatment could not be adequately estimated. Even when these two cases were included, no statistically significant differences in NRS reduction were observed (p > 0.05). However, these two cases indicate that EMS physician care as a back-up strategy can help to improve quality of care over paramedic care, even in cases in which teleconsultation alone might not be sufficient.

## Conclusions

Our findings suggest that routine remote physician-based telemedically-delegated (opioid-based) analgesia in trauma and non-trauma emergencies, as applied by paramedics, shows comparable efficacy to analgesia administered by on-scene prehospital EMS physicians. In the present study, the analgesic quality achieved in both groups was noticeably above the required minimum standard. No patient safety concerns occurred in cases with telemedically-delegated analgesia.

Mean mission duration is significantly shorter in teleconsultation cases, so that a physician can support more patients in a shorter time and in a wider radius. As it can take time for prehospital EMS physicians to arrive on-site and necessary patient treatment might therefore be delayed, the teleconsultation system bears the potential to ensure timely analgesia, even in areas underserved by EMS physicians. This depicts an improvement in the quality of patient treatment.

In the future, the transferability of the telemedical concept to other regions and countries should be investigated. Thereafter, the findings should be confirmed by future multicentre trials, which should include larger patient samples and preferably use a prospective study design with randomized allocation to telemedically-supported versus on-site physician-based analgesia.

## References

[1] Neugebauer EAM (2012). The treatment of patients with severe and multiple traumatic injuries. Dtsch. Arzteblatt Int.

[2] Jennings PA, Cameron P, Bernard S (2011). Epidemiology of prehospital pain: an opportunity for improvement. Emerg. Med. J. EMJ.

[3] Pierik JGJ (2015). Pain management in the emergency chain: the use and effectiveness of pain management in patients with acute musculoskeletal pain. Pain Med. Malden Mass.

[4] Albrecht E (2013). Undertreatment of acute pain (oligoanalgesia) and medical practice variation in prehospital analgesia of adult trauma patients: a 10 yr retrospective study. Br. J. Anaesth..

[5] Carr DB, Goudas LC (1999). Acute pain. Lancet Lond. Engl.

[6] Chambers JA, Guly HR (1993). The need for better pre-hospital analgesia. Arch. Emerg. Med..

[7] Stork, B. & Hofmann-Kiefer, K. Analgesia as an important component of emergency care. *Anaesthesist* **58**, 639–48, quiz 649–650, doi:10.1007/s00101-009-1585-1 (2009). 10.1007/s00101-009-1585-1 19562402

[8] Nikolaou NI (2015). Part 5: Acute coronary syndromes: 2015 International Consensus on Cardiopulmonary Resuscitation and Emergency Cardiovascular Care Science with Treatment Recommendations. Resuscitation.

[9] Priori SG (2015). ESC Guidelines for the management of patients with ventricular arrhythmias and the prevention of sudden cardiac death: The Task Force for the Management of Patients with Ventricular Arrhythmias and the Prevention of Sudden Cardiac Death of the European Society of Cardiology (ESC). Endorsed by: Association for European Paediatric and Congenital Cardiology (AEPC). Eur. Heart J..

[10] Roffi M (2016). ESC Guidelines for the management of acute coronary syndromes in patients presenting without persistent ST-segment elevation: Task Force for the Management of Acute Coronary Syndromes in Patients Presenting without Persistent ST-Segment Elevation of the European Society of Cardiology (ESC). Eur. Heart J..

[11] Benditz A (2016). Prospective medium-term results of multimodal pain management in patients with lumbar radiculopathy. Sci. Rep.

[12] Brokmann JC (2016). Analgesia by telemedically supported paramedics compared with physician-administered analgesia: A prospective, interventional, multicentre trial. Eur. J. Pain Lond. Engl..

[13] Friesgaard KD (2016). Efficacy and safety of intravenous fentanyl administered by ambulance personnel. Acta Anaesthesiol. Scand..

[14] Jennings PA, Lord B, Smith K (2015). Clinically meaningful reduction in pain severity in children treated by paramedics: a retrospective cohort study. Am. J. Emerg. Med..

[15] SQR-BW, B W. Available at http://www.sqrbw.de/docs/7-3_Schmerzreduktion.pdf. Accessed 22.05.2016 (2016).

[16] Wilson JE, Pendleton JM (1989). Oligoanalgesia in the emergency department. Am. J. Emerg. Med..

[17] Bundesärztekammer. Indikationskatalog für den Notarzteinsatz: Handreichung für Telefondisponenten in Notdienstzentralen und Rettungsleitstellen. *Dtsch Arztebl Int*. **110**, A–521 (2013).

[18] Bergrath S (2013). Implementation phase of a multicentre prehospital telemedicine system to support paramedics: feasibility and possible limitations. Scand. J. Trauma Resusc. Emerg. Med.

[19] Bergrath S, Rossaint R, Lenssen N, Fitzner C, Skorning M (2013). Prehospital digital photography and automated image transmission in an emergency medical service - an ancillary retrospective analysis of a prospective controlled trial. Scand. J. Trauma Resusc. Emerg. Med.

[20] Brokmann JC (2015). [Potential and effectiveness of a telemedical rescue assistance system. Prospective observational study on implementation in emergency medicine]. Anaesthesist.

[21] Sanchez-Ross M (2011). The STAT-MI (ST-Segment Analysis Using Wireless Technology in Acute Myocardial Infarction) trial improves outcomes. JACC Cardiovasc. Interv.

[22] Sejersten M (2008). Effect on treatment delay of prehospital teletransmission of 12-lead electrocardiogram to a cardiologist for immediate triage and direct referral of patients with ST-segment elevation acute myocardial infarction to primary percutaneous coronary intervention. Am. J. Cardiol..

[23] Kim KM (2015). Efficacy of a New Medical Information system, Ubiquitous Healthcare Service with Voice Inception Technique in Elderly Diabetic Patients. Sci. Rep.

[24] Rortgen D (2013). Comparison of physician staffed emergency teams with paramedic teams assisted by telemedicine–a randomized, controlled simulation study. Resuscitation.

[25] Fehn K (2014). Strafbarkeitsrisiken für Notärzte und Aufgabenträger in einem Telenotarztsystem. MedR.

[26] Christian Katzenmeier & Stefania Schrag-Slavu. *Rechtsfragen des Einsatzes der Telemedizin im Rettungsdienst*. (Springer-Verlag, 2010).

[27] von Elm E (2007). The Strengthening the Reporting of Observational Studies in Epidemiology (STROBE) statement: guidelines for reporting observational studies. PLoS Med..

[28] Felzen M (2016). Improved technical performance of a multifunctional prehospital telemedicine system between the research phase and the routine use phase - an observational study. J. Telemed. Telecare.

[29] Middleton PM (2010). Effectiveness of morphine, fentanyl, and methoxyflurane in the prehospital setting. Prehospital Emerg. Care Off. J. Natl. Assoc. EMS Physicians Natl. Assoc. State EMS Dir.

[30] Bendall JC, Simpson PM, Middleton PM (2011). Prehospital analgesia in New South Wales, Australia. Prehospital Disaster Med.

[31] Walsh B, Cone DC, Meyer EM, Larkin GL (2013). Paramedic attitudes regarding prehospital analgesia. Prehospital Emerg. Care Off. J. Natl. Assoc. EMS Physicians Natl. Assoc. State EMS Dir.

[32] Reimann B, Maier BC, Lott R, Konrad F (2004). Gefährdung der Notarztversorgung im ländlichen Gebiet. Notfall Rettungsmed.

[33] Schmiedel, R & Behrendt, H. Leistungen des Rettungsdienstes 2008/09. Analyse des Leistungsniveaus im Rettungsdienst für die Jahre 2008 und 2009. Bundesanstalt für Straßenwesen (Hrsg.): Berichte der Bundesanstalt für Straßenwesen. Mensch und Sicherheit (Wirtschaftsverlag NW, Bremerhaven, 2011).

[34] Corrigan M, Wilson SS, Hampton J (2015). Safety and efficacy of intranasally administered medications in the emergency department and prehospital settings. Am. J. Health-Syst. Pharm. AJHP Off. J. Am. Soc. Health-Syst. Pharm..

[35] Jennings PA (2012). Morphine and ketamine is superior to morphine alone for out-of-hospital trauma analgesia: a randomized controlled trial. Ann. Emerg. Med..

[36] Bloemhoff A (2016). Solo emergency care by a physician assistant versus an ambulance nurse: a cross-sectional document study. Scand. J. Trauma Resusc. Emerg. Med.

